# Effects of Changing Body Weight Distribution on Mediolateral Stability Control during Gait Initiation

**DOI:** 10.3389/fnhum.2017.00127

**Published:** 2017-03-27

**Authors:** Teddy Caderby, Eric Yiou, Nicolas Peyrot, Xavier de Viviés, Bruno Bonazzi, Georges Dalleau

**Affiliations:** ^1^Laboratoire IRISSE, UFR des Sciences de l’Homme et de l’Environnement, Université de la RéunionIle de la Réunion, France; ^2^Laboratoire CIAMS, Université Paris Sud, Université Paris-SaclayOrsay, France; ^3^Laboratoire CIAMS, Université d’OrléansOrléans, France

**Keywords:** anticipatory postural adjustments, postural stability, gait initiation, external load, weight bearing asymmetry, margin of stability, balance

## Abstract

During gait initiation, anticipatory postural adjustments (APA) precede the execution of the first step. It is generally acknowledged that these APA contribute to forward progression but also serve to stabilize the whole body in the mediolateral direction during step execution. Although previous studies have shown that changes in the distribution of body weight between both legs influence motor performance during gait initiation, it is not known whether and how such changes affect a person’s postural stability during this task. The aim of this study was to investigate the effects of changing initial body weight distribution between legs on mediolateral postural stability during gait initiation. Changes in body weight distribution were induced under experimental conditions by modifying the frontal plane distribution of an external load located at the participants’ waists. Fifteen healthy adults performed a gait initiation series at a similar speed under three conditions: with the overload evenly distributed over both legs; with the overload strictly distributed over the swing-limb side; and with the overload strictly distributed over the stance-leg side. Our results showed that the mediolateral location of center-of-mass (CoM) during the initial upright posture differed between the experimental conditions, indicating modifications in the initial distribution of body weight between the legs according to the load distribution. While the parameters related to the forward progression remained unchanged, the alterations in body weight distribution elicited adaptive changes in the amplitude of APA in the mediolateral direction (i.e., maximal mediolateral shift of the center of pressure (CoP)), without variation in their duration. Specifically, it was observed that the amplitude of APA was modulated in such a way that mediolateral dynamic stability at swing foot-contact, quantified by the margin of stability (i.e., the distance between the base of support boundary and the extrapolated CoM position), did not vary between the conditions. These findings suggest that APA seem to be scaled as a function of the initial body weight distribution between both legs so as to maintain optimal conditions of stability during gait initiation.

## Introduction

Gait initiation, which corresponds to the transition from an upright stance to walking, is a locomotor task that is frequently executed in daily life. This task can be decomposed into two successive phases: a “postural phase”, which precedes the swing heel-off time, followed by a “step execution phase” (Brenière et al., 1987; Brunt et al., 1999). During the postural phase, dynamic phenomena known as “anticipatory postural adjustments” (APA) are developed along the progression (or anteroposterior) axis (Brenière et al., 1987; Crenna and Frigo, 1991). These APA are manifested by a backwards shift in the center of pressure (CoP), which acts to propel the center of mass (CoM) forwards. It is acknowledged that these anticipatory dynamic phenomena create the conditions that are needed to reach the intended gait speed at the end of the first step (Brenière et al., 1987; Lepers and Brenière, 1995; Michel and Do, 2002).

APA are also described along the mediolateral axis. They are characterized by a CoP shift towards the swing-leg side, which propels the CoM towards the stance-leg side prior to swing foot-off (Jian et al., 1993; Elble et al., 1994). These postural dynamics are known to be crucial for stabilizing the whole body during step execution (McIlroy and Maki, 1999; Rogers et al., 2001; Yiou et al., 2012a). Indeed, the act of lifting the swing foot may create a mediolateral “gap” between the CoM and the CoP, which is then located in a new position beneath the stance foot. This gap may be responsible for a disequilibrium torque, which accelerates the CoM towards the swing-leg side and can potentially lead to a sideways fall. During gait initiation, this disequilibrium torque is invariably attenuated by the CoM displacement towards the stance leg-side during APA. Mediolateral APA are thus generally considered a feed-forward mechanism; one that is responsible for controlling mediolateral stability during gait initiation (McIlroy and Maki, 1999; Mille et al., 2014). Nevertheless, it is noteworthy that the mediolateral swing-foot placement (i.e., step width) may also be modulated in order to control mediolateral stability during gait initiation (Zettel et al., 2002a,b; Caderby et al., 2014). Modulating the swing-foot placement allows the CoM to be repositioned inside the base of support, thus ensuring postural stability.

The question of whether and how the initial body weight distribution between both legs may influence the gait initiation process in able-bodied subjects has been addressed in recent studies (Patchay and Gahéry, 2003; Azuma et al., 2007). In these studies, weight distribution between both legs were experimentally modified by asking subjects to shift their weight either onto the stance leg-side or swing leg-side prior to gait initiation, thus yielding an asymmetrical body weight distribution between the legs. Overall, these studies reported that an increase in the weight distribution over the swing leg-side induced APA of a longer duration, a shorter duration of step execution, and faster forward progression velocity compared with gait initiation performed with a posture with symmetrical body weight distribution over both legs. This effect was reversed when subjects shifted their weight over the stance leg-side. Despite the efforts made by the aforementioned authors, there is still an overall lack of understanding of how initial body weight distribution between the legs influences mediolateral postural stability during gait initiation. Such knowledge would be particularly significant for the prevention of falls, because mediolateral instability is known to be responsible for sideways falls and serious hip fractures (Nevitt and Cummings, 1993; Kannus et al., 2006).

When body weight is positioned closer to the stance leg-side in the initial upright posture, the amplitude of the mediolateral postural dynamics generated during APA needs to be scaled down (when compared with posture with symmetrical body weight distribution) to maintain postural stability during step execution. If it is not, the CoM may be propelled beyond the base of support with the risk of an imbalance towards the stance leg-side. Conversely, when body weight is positioned closer towards the swing leg-side in the initial posture, the amplitude of the mediolateral postural dynamics during APA needs to be scaled up to maintain postural stability during step execution. If it is not, the tendency of the CoM to fall towards the swing leg-side during step execution will be exacerbated, with a potential risk of imbalance. A strategy of increasing step width may then be required to maintain balance. Thus, body weight distribution between both legs can influence mediolateral stability during gait initiation, according to the loaded limb side. Nevertheless, recent results have suggested that the central nervous system is able to modulate the stabilizing features of gait initiation, i.e., mediolateral APA and swing-foot placement, so as to maintain an invariant mediolateral stability in situations with a postural constraint, e.g., induced by a lateral arm motion (Yiou and Do, 2011), an obstacle to clear (Yiou et al., 2016b) or a faster gait speed (Caderby et al., 2014). Therefore, the question arises as to whether such adaptations occur when gait initiation is performed and body weight distribution between both legs is modified.

It should be noted that, in contrast with some specific cases of pathological patients (e.g., patients that have suffered a stroke Marigold and Eng, 2006; Tessem et al., 2007), healthy adults evenly distribute their body weight between both legs during quiet standing (Bouisset and Maton, 1995; Hill and Vandervoort, 1996). In able-bodied subjects, natural posture with asymmetrical body weight distribution between the legs can be found in ecological situations, typically when one side of the body is loaded with an additional mass (e.g., carrying an object with a single hand or a backpack on one shoulder, etc.). Some authors have reported that, under such conditions, CoM location during quiet stance is shifted towards the overloaded limb side (Wu and MacLeod, 2001; Haddad et al., 2011). To our knowledge, although recent studies have investigated the effect of changes in body weight distribution induced by load carriage and by overweight on postural control during various motor tasks (e.g., Li and Aruin, 2007, 2009; Robert et al., 2007; Cau et al., 2014; Chen et al., 2015), no study has sought to examine the effect of changes in body weight distribution between the legs induced by load carriage on postural stability during gait initiation.

Thus, the aim of this study was to investigate the effect of changes in body weight distribution between both legs induced by an external load on mediolateral stability control during gait initiation. Based on previous findings from the literature (Yiou and Do, 2011; Caderby et al., 2014; Yiou et al., 2016b), we hypothesized that healthy young adults would modulate the stabilizing features of gait initiation (i.e., mediolateral APA and/or step width) as a function of initial body weight distribution so that the mediolateral stability remains unchanged.

## Materials and Methods

### Subjects

Fifteen healthy subjects (13 males, 2 females; mean age: 21 ± 2 years, height: 176 ± 9 cm, weight: 70 ± 10 kg) participated in this experiment. All gave written consent after being fully informed of the test procedure, which was approved by the Institutional Review Board for the Protection of Human Research of the University of La Réunion and conducted in accordance with the Declaration of Helsinki.

### Experimental Set-Up and Procedure

Gait initiation was performed from a first force-plate located at the beginning of a 5-m walkway. A second force-plate was located immediately in front of this initial force-plate so that the first step naturally landed on it. The two force-plates (40 × 60 cm, AMTI, Watertown, MA, USA), embedded in the walkway, recorded the ground reaction forces and moments at 1000 Hz. Beforehand, a foot switch sensor (25 mm, Biometrics, France) was secured to the first force-plate, under the heel of the subject’s swing leg. Force-plate and foot switch signals were synchronized and transmitted to an acquisition system.

Initially, subjects were instructed to stand barefoot in a comfortable and natural upright posture with their arms alongside their trunk. They were asked to stand as still as possible and to fixate their gaze on a target placed at eye level, at a distance of 6 m. After receiving a verbal “all set” signal, subjects initiated gait on their own initiative and continued walking straight ahead to the end of the walkway. The swing leg was selected by the subject and was maintained throughout the experiment. After each trial, the subjects repositioned themselves in the standardized foot position (see McIlroy and Maki, 1997) previously marked on the first force-plate. The experimenter triggered the data acquisition when the subjects were motionless and at least 1 s before the “all set” signal.

**Figure 1:**
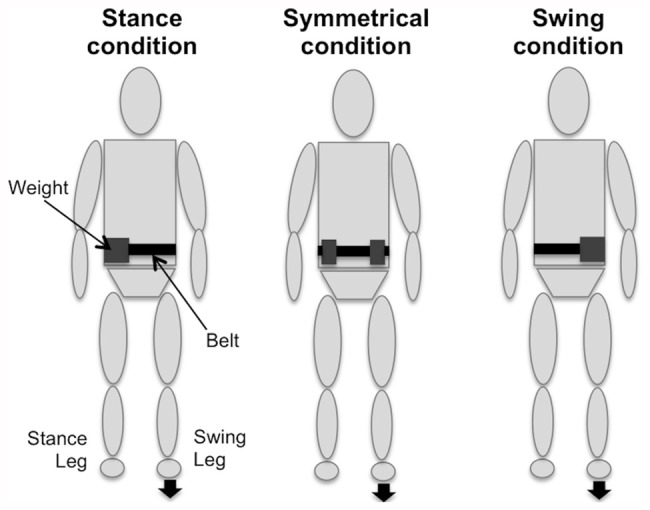
**Schematic representation of the various experimental conditions (Stance, Symmetrical and Swing).** Note that the weights were ventrally and dorsally added.

Each subject performed a gait initiation series under three experimental conditions (Figure 1): with a load symmetrically distributed around the waist (Symmetrical condition), with an asymmetrical load strictly placed over the stance-limb side (Stance condition), and with an asymmetrical load strictly placed over the swing-limb side (Swing condition). The overload consisted of a belt positioned at a height that was close to the subject’s body CoM, which in this study corresponded to 57% of the subject’s height (Winter, 1990). Weights were attached to this belt ventrally and dorsally in order to reach the desired load. The chosen mass was 10% of the subject’s body mass, because this was shown to be sufficient to modify the CoM location in quiet standing (Wu and MacLeod, 2001). In all conditions, the weights were placed symmetrically with respect to the sagittal plane so as avoid modifying the anteroposterior CoM location during the upright posture (Caderby et al., 2013a).

To enable a comparison to be made between the various experimental conditions, the subjects were instructed to maintain a similar self-selected gait speed in all conditions. The order of the experimental conditions was randomized across subjects. In each condition, the subjects performed two familiarization trials, followed by eight trials from which data were collected. The subjects rested for 3 min between each condition.

### Data Analysis

Before analysis, the force-plate signals were filtered using a low-pass Butterworth filter with a 10 Hz cut-off frequency. The anteroposterior and mediolateral CoP coordinates were calculated from force-plate data in accordance with the manufacturer’s instructions (AMTI Manual). The anteroposterior (x”CoM) and mediolateral (y”CoM) accelerations of the CoM were determined from ground reaction forces according to Newton’s second law. The anteroposterior and mediolateral CoM velocities and displacements were computed by successive numerical integrations of the corresponding acceleration using the trapezoidal rule. Calculations were performed with integration constant null, i.e., initial velocity and displacement equal to zero (Brenière et al., 1987). By convention, the CoM displacement and velocity and the CoP displacement were considered positive when directed forwards and towards the swing leg-side.

Several temporal events were determined to calculate our various dependent variables. The APA onset was detected when y”CoM deviated 2.5 standard deviations from its baseline value (Yiou et al., 2012b). Time of heel-off was detected from the foot switch sensor (Caderby et al., 2013b). The instant of swing foot-contact was determined when the vertical force signal of the second force-plate exceeded 10 N. The instant of swing foot-off was identified from the mediolateral CoP displacement (Melzer et al., 2007; Uemura et al., 2011), at the precise point when the slope of the CoP shift toward the stance leg suddenly changed (absolute CoP slope <100 mm/s, 2 samples in a row). The instant of stance foot-off was determined when the vertical force signal of the first force-plate dropped below 5 N.

### Dependent Variables

**Figure 2:**
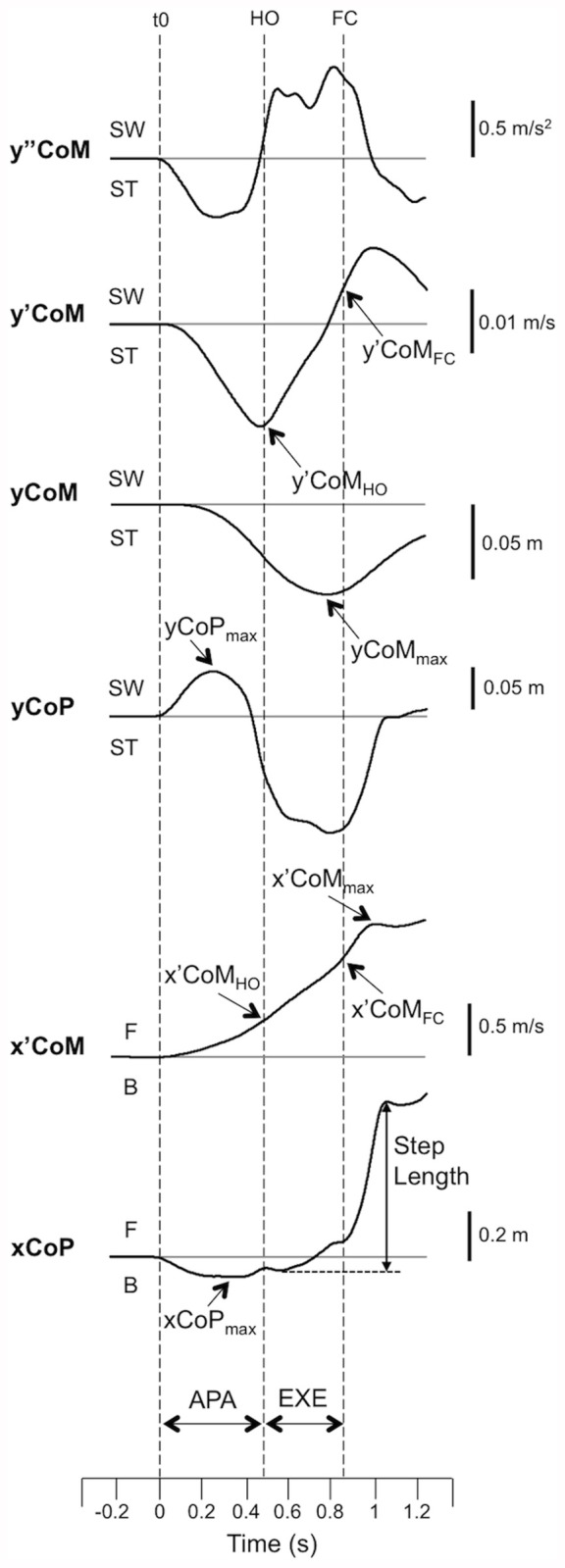
**Example of the main biomechanical traces obtained for one subject during gait initiation gait (one trial) under the Symmetrical condition.** y”CoM, y’CoM, yCOM, yCoP: mediolateral center-of-mass (CoM) acceleration, velocity and displacement, mediolateral center-of-pressure (CoP) displacement, respectively. x’COM, xCoP: anteroposterior CoM velocity and anteroposterior CoP displacement, respectively. T0 indicates the onset variation of the y”CoM trace from the baseline. HO and FC: swing heel-off and swing foot-contact, respectively. F and B indicate forward and backward movement, respectively. ST and SW indicate stance limb and swing limb, respectively. x’CoM_HO_, x’CoM_FC_, x’CoM_max_, xCoP_max_: anteroposterior CoM velocity at heel-off, at foot-contact, at the end of the first step and maximal backwards CoP shift, respectively. y’CoM_HO_, y’CoM_FC_, yCoM_max_, yCoP_max_: mediolateral CoM velocities at heel-off, at foot-contact, maximal mediolateral CoM displacement towards the stance leg and maximal mediolateral CoP displacement towards the swing leg during anticipatory postural adjustments (APA). APA and EXE: time windows for APA and step execution.

The main dependent variables are illustrated in Figure 2. Initial anteroposterior and mediolateral CoM locations were estimated by averaging respectively the anteroposterior and mediolateral CoP positions during the 250 ms period preceding the “all set” signal (McIlroy and Maki, 1999). These initial locations also served as initial constants for computing the CoM position from its displacement during the time course of the gait initiation movement.

APA duration corresponded to the delay between APA onset and the heel-off of the swing leg. Step execution duration corresponded to the time between the swing heel-off and the swing foot-contact. Anteroposterior and mediolateral APA amplitudes were characterized respectively by the maximal backwards CoP shift and the maximal mediolateral CoP shift towards the swing leg during APA. The anteroposterior and mediolateral CoM velocities to time of heel-off and foot-contact were analyzed. Both the peak of mediolateral CoM displacement towards the stance leg during gait initiation and the mediolateral distance between the CoM and the CoP at this instant were calculated. Progression velocity was quantified at the peak of the anteroposterior CoM velocity reached at the end of the first step (Brenière et al., 1987). Step length was calculated as the anteroposterior distance between the CoP position at the swing foot-off time and the stance foot-off time (Gélat et al., 2006).

The concept of “margin of stability” (MoS) introduced by Hof et al. (2005) was used to quantify mediolateral dynamic stability in the present study. The MoS corresponded to the difference between the mediolateral boundary of the base of support (BoS_ymax_) and the mediolateral position of the “extrapolated CoM” at foot-contact (YcoM_FC_), i.e., MoS = BoS_ymax_ − YcoM_FC_. As in Hof et al. (2005), BoS_ymax_ was determined from CoP data. Specifically, BoS_ymax_ was defined as the mediolateral CoP position at the time of stance foot-off, which at this point was located beneath the swing-foot (Hof et al., 2005). The mediolateral distance between the CoP position at this time (i.e., stance foot-off) and the mean CoP position over the single stance period of the leg stance represented the step width, and was representative of the size of the mediolateral base of support.

Based on the study of Hof et al. (2005), the mediolateral position of the extrapolated CoM at foot-contact (YcoM_FC_) was calculated as follows:

$${\text{YcoM}}_{\text{FC}}\;=\;{\text{yCoM}}_{\text{FC}}+\frac{{\text{y'CoM}}_{\text{FC}}}{{\text{ω}}_{0}},$$

where yCoM_FC_ and y’CoM_FC_ are respectively the mediolateral CoM position and velocity at foot-contact, and ω_0_ is the eigenfrequency of the body modeled as an inverted pendulum, calculated as:

$${\text{ω}}_{0}\;=\;\sqrt{\frac{g}{l},}$$

where *g* = 9.81 m/s^2^ is the gravitational acceleration and *l* is the length of the inverted pendulum, which in this study corresponded to 57.5% of the body height (Winter, 1990).

Mediolateral dynamic stability at foot-contact is ensured on condition that YcoM_HC_ is within BoS_ymax_, which corresponds to a positive MoS. A negative MoS indicates mediolateral instability and implies that a corrective action (e.g., in the form of an additional lateral step) has to be undertaken to maintain balance.

### Statistical Analysis

Mean and standard deviation values for each dependent variable were calculated over the eight trials performed in each experimental condition. Repeated measures ANOVA with the load distribution condition (Stance, Symmetrical and Swing) as within-subject factors were conducted on each of these variables in turn. For each ANOVA, partial eta-squared value ($\eta_{\text{p}}^{2}$) was presented as a measure of effect size. When a significant statistical difference was found, *post hoc* comparisons were performed using pairwise comparisons with a Holm-Bonferroni correction (Holm, 1979). The level of significance was set at **α** = 0.05.

## Results

### Description of the Biomechanical Traces

Gait initiation movement globally followed a similar pattern under the various load distribution conditions. This pattern is illustrated in Figure 2. The heel-off of the swing leg was systematically preceded by postural dynamics that corresponded to APA. During APA, CoP shifted backwards and laterally towards the swing leg. In the mediolateral direction, CoP displacement reached a peak value towards the swing leg, while CoM displacement and velocity were directed towards the stance leg. The mediolateral CoM velocity trace reached the first peak value towards the stance-leg side at around heel-off. This trace then dropped towards the swing-leg side. The second peak value towards this side was reached a few milliseconds after foot-contact. The CoM displacement reached a peak value towards the stance-leg side during the execution phase. The CoM then fell towards the swing-leg side. In the anteroposterior direction, the CoM velocity increased progressively until it reached a peak value a few milliseconds after foot-contact.

### Initial Posture

Load distribution significantly affected the initial CoM location in the mediolateral direction *F*_(1,14)_ = 73.34, *P* < 0.001, $\eta_{\text{p}}^{2}$ = 0.84), but not along the anteroposterior direction (*P* > 0.05, $\eta_{\text{p}}^{2}$ = 0.02). *Post hoc* analysis revealed that the initial mediolateral CoM location differed significantly between the three experimental conditions (*P* < 0.001). In the Symmetrical condition, the initial mediolateral CoM location was 0.1 ± 0.7 cm on the swing-leg side with respect to the midline between both feet. This initial mediolateral CoM position (with respect to the midline between both feet) was significantly closer to the swing leg in the Swing condition (0.8 ± 0.7 cm at the swing-leg side), and significantly closer to the stance leg in the Stance condition (0.8 ± 0.8 cm at the stance-leg side) compared with the Symmetrical condition.

### Anticipatory Postural Adjustments

**Figure 3:**
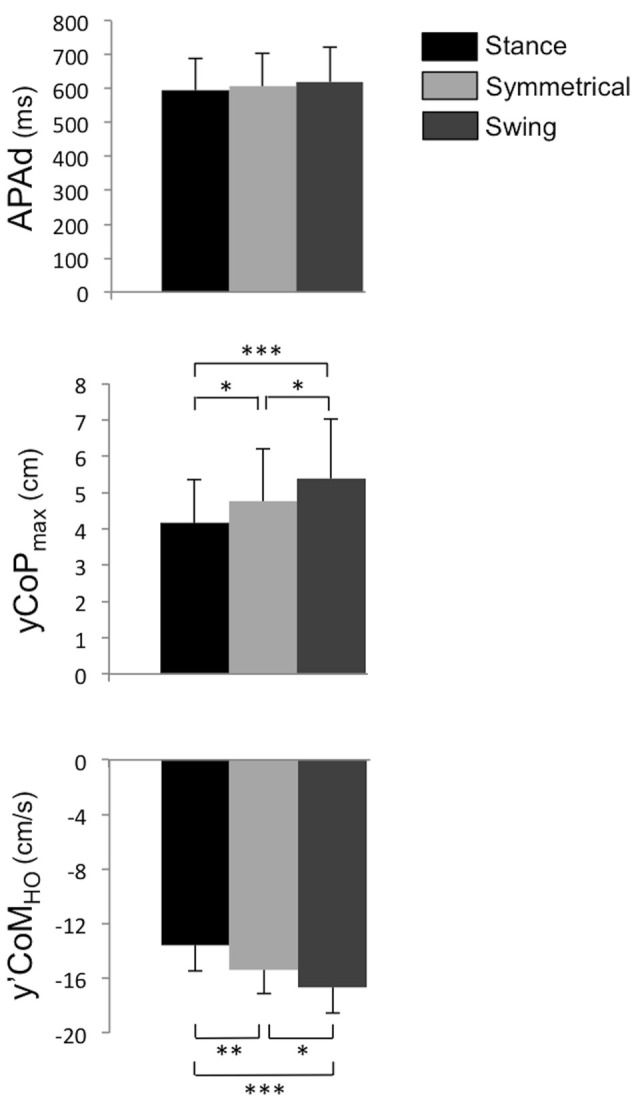
**Mean of temporo-spatial features of APA under the Stance, Symmetrical and Swing conditions.** APAd: APA duration; yCoP_max_: peak of mediolateral CoP shift towards the swing leg during APA. y’CoM_HO_: mediolateral CoM velocity at heel-off. Negative values indicate a displacement or velocity directed towards the stance leg. *,**,***Significant difference with *P* < 0.05, *P* < 0.01 and *P* < 0.001, respectively.

**Figure 4:**
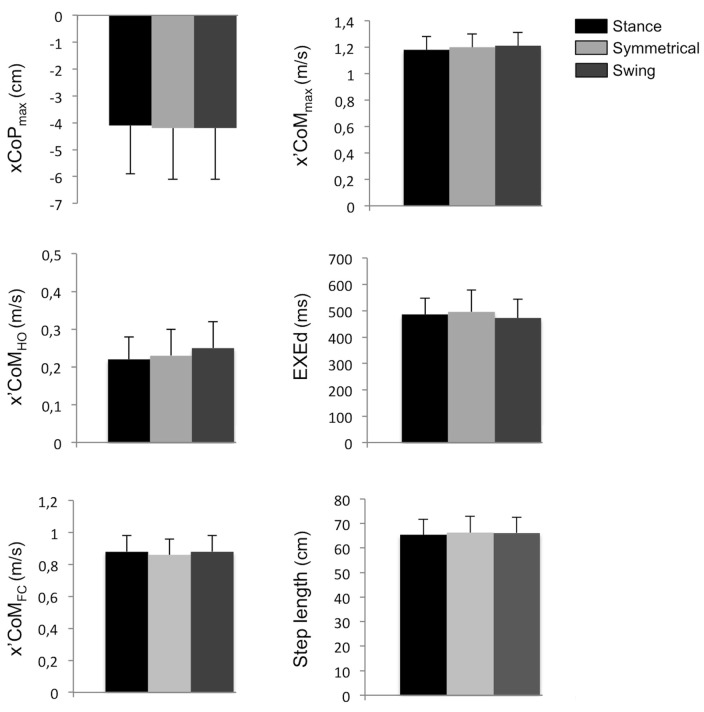
**Mean of parameters related to the forward progression under the Stance, Symmetrical and Swing conditions.** xCoP_max_: maximal backward shift of the CoP during APA. x’CoM_HO_: anteroposterior velocity of the CoM at heel-off. x’CoM_FC_: anteroposterior CoM velocity at foot-contact. x’CoM_max_: peak of the anteroposterior CoM velocity at the end of the first step. EXEd: duration of step execution. Negative values indicate a displacement or velocity directed backwards.

No effect of the load distribution was found on APA duration (*P* > 0.05, $\eta_{\text{p}}^{2}$ = 0.17, Figure 3). Similarly, statistical analysis indicated that the load distribution did not affect the maximal backward CoP shift (*P* > 0.05, $\eta_{\text{p}}^{2}$ = 0.02, Figure 4) and the anteroposterior CoM velocity at heel-off (*P* > 0.05, $\eta_{\text{p}}^{2}$ = 0.05, Figure 4), indicating that the anticipatory postural dynamics in the anteroposterior direction were not modified by changes in body weight distribution. In contrast, with regard to the mediolateral postural dynamics, a significant effect of the load distribution was found for the peak of the mediolateral CoP shift during APA (*F*_(1,14)_ = 13.46, *P* < 0.001, $\eta_{\text{p}}^{2}$ = 0.49) and the mediolateral CoM velocity at heel-off (*F*_(1,14)_ = 20.42, *P* < 0.001, $\eta_{\text{p}}^{2}$ = 0.59). *Post hoc* analysis indicated that these parameters differed significantly between all of the conditions (Figure 3). More specifically, when compared with the Symmetrical condition, these parameters were significantly higher in the Swing condition, and lower in the Stance condition.

### Mediolateral Stability

**Figure 5:**
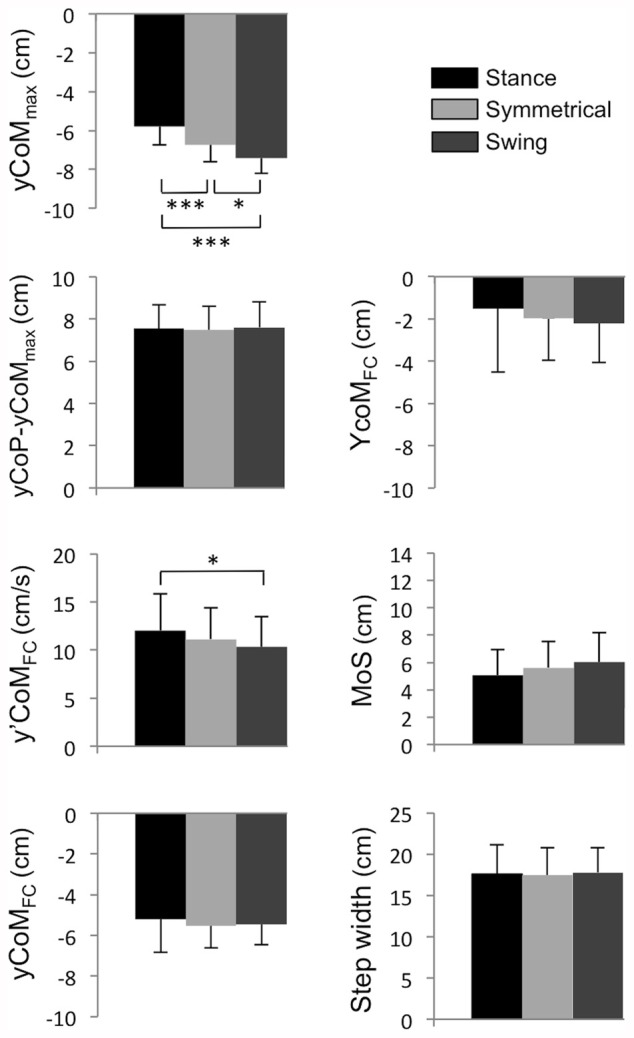
**Mean of parameters related to the mediolateral stability under the Stance, Symmetrical and Swing conditions.** yCoM_max_: maximal CoM displacement towards the stance leg. yCoP-yCoM_max_: mediolateral distance between CoP and CoM at the instant when the CoM reaches its maximal displacement towards the stance leg. y’CoM_FC_: mediolateral CoM velocity at foot-contact. yCoM_FC_: mediolateral CoM position at the time of foot-contact. YcoM_FC_: mediolateral extrapolated CoM position at the time of foot-contact. Margin of stability (MoS). Negative values indicate a displacement or velocity directed towards the stance leg. *,***Significant difference with *P* < 0.05 and *P* < 0.001, respectively.

A significant effect of the load distribution was found for the maximal mediolateral CoM displacement towards the stance leg during step execution (*F*_(1,14)_ = 22.53, *P* < 0.001, $\eta_{\text{p}}^{2}$ = 0.62). Compared with the Symmetrical condition, this parameter was significantly higher in the Swing condition, and significantly lower in the Stance condition (Figure 5). Despite these variations, both the mediolateral CoP position (*P* > 0.05, $\eta_{\text{p}}^{2}$ = 0.10) and the mediolateral gap between the CoP and CoM at the time of maximal mediolateral CoM displacement were unchanged in all of the conditions (*P* > 0.05, $\eta_{\text{p}}^{2}$ = 0.13, Figure 5). A significant effect of the load distribution was also found for the mediolateral CoM velocity at foot-contact (*F*_(1,14)_ = 4.65, *P* < 0.05, $\eta_{\text{p}}^{2}$ = 0.25). Specifically, the mediolateral CoM velocity was significantly higher in the Stance condition than in the Swing condition (Figure 5), whilst the *post hoc* analysis revealed no other difference. Finally, there was no effect of load distribution on the mediolateral CoM position at foot-contact (*P* > 0.05, $\eta_{\text{p}}^{2}$ = 0.06, Figure 5), the mediolateral position of the extrapolated CoM at foot-contact (*P* > 0.05, $\eta_{\text{p}}^{2}$ = 0.07, Figure 5), the margin of stability (*P* > 0.05, $\eta_{\text{p}}^{2}$ = 0.14, Figure 5), and step width (*P* > 0.05, $\eta_{\text{p}}^{2}$ = 0.03, Figure 5).

### Motor Performance

Our results showed that the load distribution had no effect on the parameters related to forward progression (Figure 4): the anteroposterior CoM velocity at foot-contact (*P* > 0.05, $\eta_{\text{p}}^{2}$ = 0.17), the peak of the anteroposterior CoM velocity at the end of the first step (*P* > 0.05, $\eta_{\text{p}}^{2}$ = 0.08), the duration of step execution (*P* > 0.05, $\eta_{\text{p}}^{2}$ = 0.15), and the step length (*P* > 0.05, $\eta_{\text{p}}^{2}$ = 0.13).

## Discussion

The aim of this study was to investigate the effects of changing the initial body weight distribution between both legs on the control of mediolateral dynamic stability during gait initiation. Changes in body weight distribution were experimentally induced by modifying the distribution along the frontal plane of an external load located at the participants’ waists.

### External Load Induced Changes in Body Weight Distribution between Both Legs

The changes in body weight distribution induced by the external load were attested by the significant differences observed in the initial mediolateral CoM location between the various experimental conditions. In the Symmetrical condition, the CoM was almost located at the midline between both feet (0.1 cm from the swing leg), reflecting a quasi-symmetrical body weight distribution between both legs. In the asymmetrical loading conditions (Stance and Swing conditions), we observed that the initial CoM location was significantly shifted towards the overloaded leg side, which is in accordance with previous studies on quiet standing (Wu and MacLeod, 2001; Haddad et al., 2011). This indicates an increase in the body weight distribution over the overloaded leg side. To be precise, the CoM was displaced by ~0.8 cm on the overloaded side (stance or swing leg) compared with the Symmetrical condition. This finding is in accordance with the deviations observed in the study by Wu and MacLeod (2001) for an asymmetrical load of 10% of body weight (≈1 cm in this previous study). The initial anteroposterior CoM location, in contrast, was not affected by changes in the external load distribution. This suggests that, in the present study, the distribution of body weight along the sagittal plane did not change between the various experimental conditions.

### Effects of the Changes in Body Weight Distribution on Mediolateral Dynamic Stability Control

In accordance with our hypothesis, our results showed that the mediolateral dynamic stability at swing foot-contact, quantified by the margin of stability, was unaffected by the changes in the initial body weight distribution. This finding suggests that the subjects developed adaptive postural strategies in order to reach an equivalent mediolateral stability when the initial body weight distribution was modified. It has been shown that mediolateral stability during gait initiation is mainly regulated by the mediolateral APA and the mediolateral swing foot-placement, i.e., the step width (McIlroy and Maki, 1999; Caderby et al., 2014; Yiou et al., 2016a). In the present study, the step width was not modified between the various experimental conditions. This implies that other forms of postural adaptations occurred in order to maintain an invariant mediolateral dynamic stability when the body weight distribution between the legs was modified.

It is well known that mediolateral APA, often considered as a lateral thrust exerted on the ground (Mouchnino and Blouin, 2013), serve to propel the CoM towards the stance foot prior to swing foot-off. Although the CoM is never repositioned over the stance foot, mediolateral APA help to minimize the extent to which the body subsequently falls towards the swing-leg side during step execution, i.e., mediolateral instability (Jian et al., 1993; Winter, 1995; Lyon and Day, 1997; McIlroy and Maki, 1999; Rogers et al., 2001; Yiou et al., 2016a). In the present study, the results showed that APA duration, i.e., the time allocated to propel the CoM toward the stance foot, did not differ between the various conditions. In contrast, the amplitude of mediolateral APA, characterized by the peak of mediolateral CoP shift towards the swing leg during APA, varied as a function of the body weight distribution over both legs. Specifically, when compared with the Symmetrical condition, mediolateral APA amplitude increased when body weight was further distributed onto the swing leg (in the Swing condition), and decreased when body weight was further distributed onto the stance leg (Stance condition). It has been shown that this anticipatory CoP shift towards the swing leg generates the propulsive forces responsible for accelerating the CoM in the opposite direction, i.e., towards the stance leg (Brenière et al., 1987; Jian et al., 1993; Winter, 1995). Consequently, these modulations in the mediolateral amplitude of APA influenced the propulsion of the CoM towards the stance foot, as attested by the differences in mediolateral CoM velocity at heel-off and the peak of mediolateral CoM displacement towards the stance foot during step execution. To be precise, compared with the Symmetrical condition, both the displacement and velocity of the CoM directed towards the stance leg increased in the Swing condition, and decreased in the Stance condition. Similar findings were observed in previous studies that investigated the effect of body weight distribution between both legs on the gait initiation process (Patchay and Gahéry, 2003; Azuma et al., 2007). However, none of these existing studies established the link between these postural adaptations and mediolateral dynamic stability during gait initiation.

Our results suggest that mediolateral APA changes in the present study aimed to compensate for the differences in the initial CoM location during the upright posture. By modulating the mediolateral APA amplitude, the subjects were able to adapt the propulsive forces so that the CoM reached a similar position relative to the stance foot during the step execution phase. Indeed, we observed that the gap between the CoP and CoM at the time of the maximal mediolateral CoM displacement towards the stance foot during step execution did not vary. This indicates that the CoM was propelled at a similar distance from the stance foot during step execution. As a consequence, the extent that the body fell laterally towards the swing leg, reflected by the mediolateral CoM position at the time of foot-contact, did not change between the various conditions. Thus, we were able to achieve an equivalent extrapolated CoM position at foot-contact in the various conditions, although the mediolateral CoM velocity at foot-contact was slightly higher in the Stance condition than in the Swing condition. In short, these adaptive changes in the mediolateral APA avoided the eventual modulation of the step width to maintain the extrapolated CoM inside the base-of-support. In addition, they helped to maintain the mediolateral stability invariant in the various conditions. These results are in line with findings from previous studies, which have shown that healthy subjects are able to modulate the features of APA in order to maintain an unchanged mediolateral stability when confronted with postural perturbation (Yiou et al., 2012a, 2016b; Caderby et al., 2014). Furthermore, these results reinforce the hypothesis that the extrapolated CoM could be a robust parameter for human balance control (Hasson et al., 2008; Yiou et al., 2012a; Caderby et al., 2014).

Our findings, which show that APA are scaled as a function of body weight distribution between the legs, are congruent with findings from the existing literature on gait initiation (Patchay and Gahéry, 2003; Azuma et al., 2007) for a task that combines stepping and pointing (Robert et al., 2007), and on a leg flexion task (Mille and Mouchnino, 1998). Recent data has provided evidence that cutaneous mechanoreceptors in the sole of the foot may be involved in the setting of APA associated with gait initiation (Mouchnino and Blouin, 2013). These receptors are sensitive to changes in pressure plantar distribution (Kavounoudias et al., 1998), which may be induced by modifications in the body weight distribution between both legs. Thus, it may be assumed that the adaptive APA changes observed in our study were associated with the integrity of these receptors in our healthy young subjects. Further investigations are required to determine whether the present results are applicable in populations that suffer from a decline in the sensitivity of these plantar receptors, such as elderly people (Wells et al., 2003; Perry, 2006). Specifically, future studies should investigate whether, like young healthy adults, the elderly are able to adapt the APA to changes in body weight distribution between the legs so as to maintain unchanged the mediolateral stability during gait initiation. Such studies may allow to identify abnormalities in the generation of APA under conditions requiring postural adaptation and may thus offer a better understanding of the causes of the frequent falls that occur among the elderly during gait initiation (Robinovitch et al., 2013).

### Effects of the Changes in Body Weight Distribution on Motor Performance

Our results indicate that changes in body weight distribution between both legs did not influence the temporal (i.e., duration of APA and step execution) and spatial (i.e., amplitude of anteroposterior APA, step length and anteroposterior CoM velocities at the various selected events) variables related to forward progression. These results are in marked contrast with those from previous studies (Patchay and Gahéry, 2003; Azuma et al., 2007; Dalton et al., 2011), which noted changes in these variables as a function of body weight distribution between both legs. This discrepancy may be explained by the fact that, contrary to these previous studies, the participants of the present study were instructed to maintain a similar progression velocity under the various experimental conditions, as attested by the absence of change in the peak of anteroposterior CoM velocity at the end of the first step. Thus, in our study, it may be assumed that the subjects scaled the temporo-spatial features of gait initiation in such way that forward progression velocity remained equivalent across all conditions. These results support the hypothesis that young healthy adults are able to independently create the conditions for both forward progression and mediolateral stability during gait initiation (Caderby et al., 2014). Interestingly, a recent study has shown that modifications in the body weight distribution over the legs along the anteroposterior direction led to scaling of the amplitude of anteroposterior APA without consistent changes in the amplitude of mediolateral APA (Hansen et al., 2016). These findings suggest that changes in the body weight distribution along the anteroposterior direction might affect the biomechanical organization of gait initiation in that direction, but not in the mediolateral direction. Further investigation is however required to confirm this hypothesis.

### Conclusion

The results of the present study highlight that young healthy adults are able to modulate mediolateral APA so as to maintain optimal conditions of dynamic stability during gait initiation with the modification of the initial body weight distribution between both legs. Bearing in mind the fact that elderly people’s falls frequently occur during gait initiation, the present findings may provide a basis for future studies that aim to better understand the mechanisms of falls in this population.

## Author Contributions

TC, EY and GD designed the study. TC, GD and BB collected the data. TC, EY, NP, XV, BB and GD analyzed and interpreted the data, drafted the manuscript and gave final approval.

## Funding

This work was supported by a grant from the University of La Reunion. The present study was funded by the French government.

## Conflict of Interest Statement

The authors declare that the research was conducted in the absence of any commercial or financial relationships that could be construed as a potential conflict of interest.
